# Advances in plant metabolic engineering through synthetic biology

**DOI:** 10.3389/fpls.2026.1957203

**Published:** 2026-09-28

**Authors:** Hind Alsuwat, Derry Alvarez, Salim Al-Babili

**Affiliations:** 1 The BioActives Lab, King Abdullah University of Science and Technology (KAUST), Thuwal, Saudi Arabia; 2 Biological and Environmental Science and Engineering (BESE) Division, King Abdullah University of Science and Technology (KAUST), Thuwal, Saudi Arabia

**Keywords:** CRISPR, genome edited plants, plant metabolic engineering, plant transformation, synthetic biology

## Abstract

The challenges associated with the escalating global population and climate change in crop production and food security are substantial. This underscores the necessity for improvements in crop nutrition, yield, and resilience to environmental stresses. Plant metabolic pathways directly influence growth, nutrient accumulation, and stress responses, rendering them prime targets for crop enhancement. Advances in plant synthetic biology and gene editing are facilitating more precise modifications of genes, regulatory elements, and metabolic pathways. This review highlights the primary technologies used in plant metabolic engineering and focuses on representative examples of their implementation for crop improvement, including but not limited to CRISPR-Cas systems, multiplex genome editing, knock-in and knock-out strategies, CRISPR-mediated gene regulation (CRISPRi/CRISPRa), base editing, prime editing, RNA targeting technologies, synthetic promoters, and gene stacking strategies. Furthermore, the emerging role of artificial intelligence in the design and optimization of metabolic pathways and genome editing tools will be briefly discussed.

## 1 Introduction

Global food security is increasingly challenged by a growing population, climate change, and widespread micronutrient deficiencies (Tilman et al., 2011). With the global population projected to reach approximately 9.8 billion by 2050, food demand is expected to increase by 30–60% (Fao, 2018; Van Dijk et al., 2021). Hence, there is a great need for crops that are resilient to environmental stresses, such as drought, salinity, and heat stress (Lesk et al., 2016). Additionally, micronutrient deficiencies affect over two billion people globally, particularly in developing countries, making biofortification of staple crops an urgent priority (Bouis and Welch, 2010). Furthermore, plants are the primary source of high-value pharmaceutical molecules (Buyel et al., 2017; Schillberg et al., 2019). Together, these factors highlight the need for crop improvement.

Conventional breeding has long addressed these challenges by selecting for phenotypic traits derived from natural genetic variation. However, its efficiency is low, as genetic variability and environmental factors increase unpredictability (Krishna et al., 2023). Marker-assisted selection (MAS), another approach that uses molecular markers to select superior plant lines with desirable traits, was developed to improve breeding accuracy by directly tracking genes of interest independent of environmental influences (Krishna et al., 2023). Nevertheless, MAS is limited by marker reliability and traits controlled by one or a few major genes (Bhat et al., 2016). Crop yield, resilience to abiotic stress, and enhanced nutritional quality are genetically and physiologically interrelated traits that rely heavily on interactions among metabolic pathways and the accumulation of specific metabolites (Huang and Dudareva, 2023).

Central to these interconnected traits are the metabolites themselves. Plant metabolites can be classified into two broad categories: (1) primary metabolites essential for cellular functions, energy production, and the synthesis of hormones and proteins, and (2) specialized metabolites (SMs) involved in growth, defense, and environmental responses (Zhu et al., 2023). SMs influence traits such as color, pollinator attraction, and responses to abiotic and biotic stress (Hounsome et al., 2008). Beyond their biological roles, SMs have high economic value as pharmaceuticals, nutrients, flavors, perfumes, and dyes (Bai et al., 2024). However, their production is often limited by low abundance and inefficient or unsustainable chemical synthesis, driving the development of metabolic engineering strategies that improve metabolic flux and optimize entire pathways (Wu and Chappell, 2008; Nielsen and Keasling, 2016).

Plant synthetic biology, the rational design and reassembly of genetic parts to reprogram cellular metabolism, has emerged as a powerful framework for addressing these challenges. Genome engineering has evolved significantly beyond initial transgenic techniques to include precise, programmable alterations at specific genomic locations (Liu et al., 2025). Notable examples of plant metabolomic engineering demonstrate what is now achievable. In rice, enhanced carotenoid accumulation in the endosperm boosts provitamin A levels; loss-of-function of the LGS1 gene in sorghum altered the levels of strigolactone, leading to reduced Striga germination and enhanced resistance to parasitic weeds (Gobena et al., 2017). Knockout of the OsCCD7 in rice similarly suppresses strigolactone production and Striga germination (Butt et al., 2018). Overexpression of OsNCED3 in rice leads to increased endogenous abscisic acid (ABA) levels and improved responsiveness to alkaline stresses (Feng et al., 2024).

The tools enabling these metabolic interventions have expanded rapidly. CRISPR-Cas systems, in their multiple configurations, such as nuclease-active, base-editing, prime-editing, and catalytically dead variants for transcriptional control, have transformed plant metabolic engineering into a routine, highly targeted discipline. Modular cloning frameworks, synthetic promoters, RNAi-based silencing, and gene stacking strategies now facilitate the construction of complex, multi-gene pathways with adjustable outputs. While precise tuning of enzyme levels has become possible by precise control of gene expression through promoter engineering, ribosome-binding site regulation, and modification of mRNA and protein stability (Nielsen and Keasling, 2016). Efficient pathway design also requires coordinated expression of multiple genes, achieved through inducible promoters, operon-like regulatory strategies, and shared regulatory systems (Nielsen and Keasling, 2016). Most recently, artificial intelligence (AI) is beginning to close the design-build-test loop, offering the prospect of autonomous, data-driven pathway optimization at a scale previously unthinkable (Singh et al., 2025).

This mini-review assesses the principal genome-engineering tools that enable synthetic biology in plants, with particular emphasis on their use to enhance nutritional quality, stress tolerance, and crop productivity (**Figure 1**), and highlights landmark applications in metabolic pathway optimization, biofortification, and climate resilience (**Figure 1**). In addition, we discuss integrating AI-guided design as a transformative, emerging layer. We also outline the key technical and regulatory challenges that must be resolved before these strategies can be broadly adopted in elite crop varieties.

**Figure 1:**
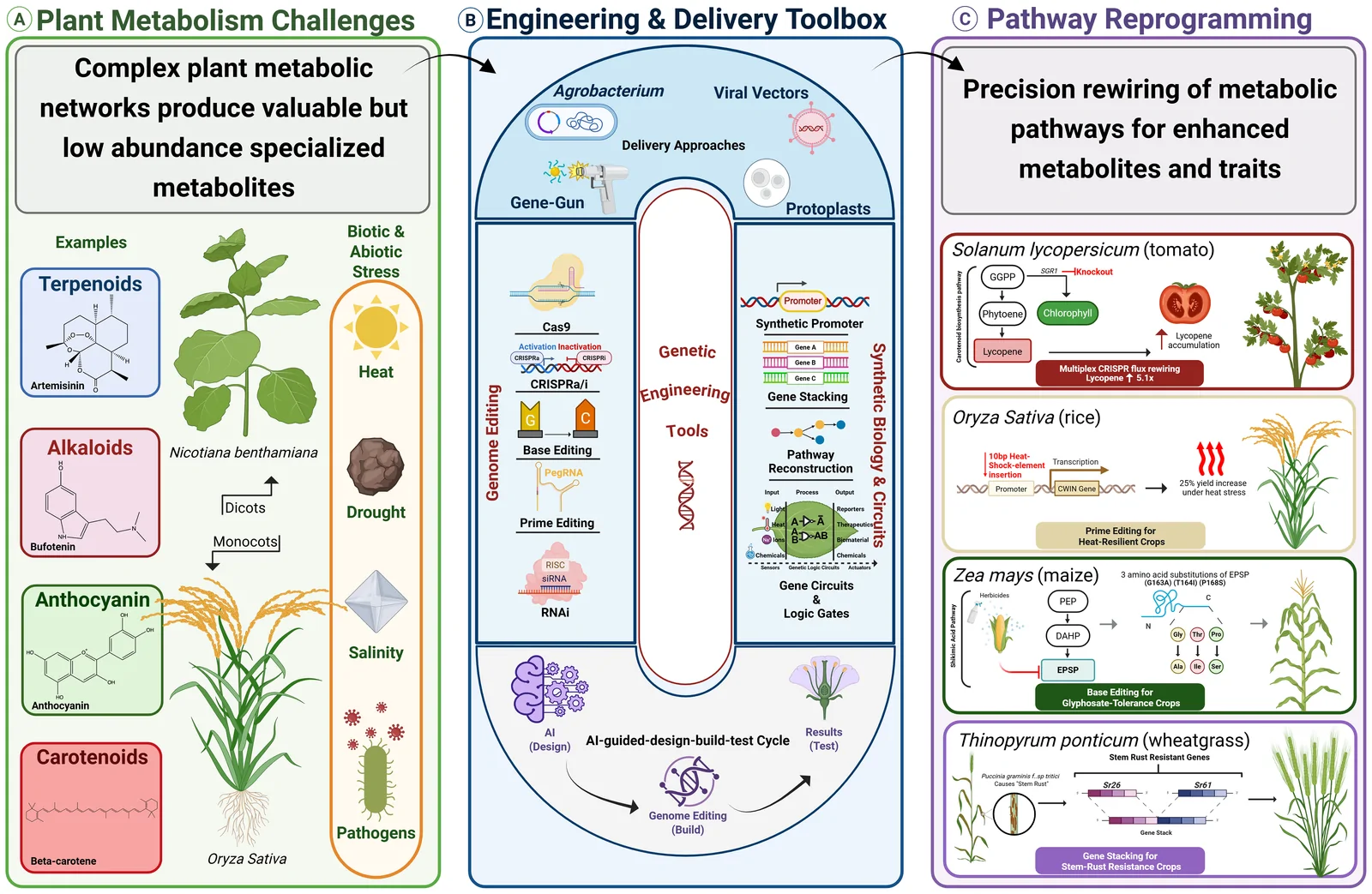
A work flow scheme in plant metabolic engineering and synthetic biology. Panel **(A)** Plant metabolic networks. Panel **(B)** Plant engineering techniques and tools. Panel **(C)** Metabolic engineering examples in different crops. This figure was generated using BioRender.

## 2 Delivery approaches in plant metabolic engineering

### 2.1 *Agrobacterium*-mediated transformation

Crown gall disease is caused by the soil bacterium Agrobacterium tumefaciens, which can transfer genetic material into plant cells (Smith and Townsend, 1907). This discovery led to plant genetic engineering, using Agrobacterium as a tool for transient and stable DNA transfer into plant tissues (White and Braun, 1941). In recombinant strains, the transfer involves two key regions: the virulence (vir) loci within the Ti plasmid, and T-DNA, which is bordered by 25 bp repeats at the left and right borders (LB and RB) (Wang et al., 1987). The ssT-DNA is transported via the Type IV secretion system, comprising about 12 proteins, and is protected by VirE2 protein in the cytoplasm (Zupan et al., 1998). In the nucleus, ssT-DNA converts to dsT-DNA, which can either remain extrachromosomal for transient expression or integrate into the genome through DSBs (Liang and Tzfira, 2013). Transformation efficiency depends on factors like tissue type, bacterial strain, and media conditions (Thiyagarajan et al., 2024). Agrobacterium-mediated transformation can insert single or low-copy genes, transfer large DNA segments (up to 10 kb), and cause minimal genomic changes (Thiyagarajan et al., 2024). It is widely used to genetically modify both dicots (e.g., Arabidopsis, tobacco, tomato, poplar, soybean) and monocots (e.g., rice, wheat, despite variable efficiency and cultivar-specific susceptibility (Herrera-Estrella et al., 2005).

### 2.2 Biolistic or particle bombardment (gene gun)

The particle bombardment concept provides a direct introduction of multiple biological materials simultaneously, either DNA, RNA, or proteins, by shooting into a high-cell-count population/explant, without cell structural disruption due to the physical distance between the treated cells and the gene gun tool (Sanford et al., 1987). This method involves mixing the DNA with inert microprojectiles, such as gold or tungsten, that physically penetrate cells at high speed and force, propelled by a pressurized gas such as helium (Sanford et al., 1987). Once inside the cell, the DNA separates from the particles and spreads throughout the cytoplasm, where it is either transiently expressed or stably integrated into the host genome (Sanford et al., 1987). Compared to other delivery systems, the particle bombardment system obviates the need to mak several selection steps to decrease or eliminate the biological vector-based system from plant tissue (Davies et al., 2013). However, depending on the amount of DNA delivered, biolistic transformation can result in a high transgene copy number, leading to uncontrolled, unpredictable insertion events, including multiple insertions, large deletions or genome rearrangements (Liu et al.,).

### 2.3 Protoplast-based delivery

Protoplasts are plant cells from which the cell walls have been enzymatically removed (Fowke and Constabel, 1985). The protoplast fusion technique has evolved into a breeding tool, enabling somatic hybridization for plant regeneration (Alexander, 2002). Protoplast fusion has been widely applied in somatic hybridization to bypass sexual incompatibility between plant species (Louzada et al., 1993). While these are great advantages of protoplast engineering, it still faces limitations due to its fragility and inability to sustain long-term culture (Aoyagi, 2011).

### 2.4 Viral vectors

Recently, plant viral vectors have become genetic engineering tools derived from natural plant viruses (Gleba et al., 2007). Lately, viral vectors have been utilized to deliver CRISPR/Cas components for virus-induced genome editing (VIGE) in model and non-model plants (Zhang et al., 2022). The viral vectors are delivered either by mechanically inoculating plants with viral particles using abrasives or by introducing viral genome constructs via Agrobacterium-mediated transformation (Gleba et al., 2007). Once the plant is infected, the virus replicates within the plant cell, enabling gene expression throughout plant tissues within a few weeks (Gleba et al., 2007). The viral vector delivery system is a fast approach that exploits the virus’s natural replication cycle, enabling independent genome amplification and high-level transgene expression (Lico et al., 2008). Despite their effectiveness, plant viral vectors have many drawbacks, including limited load capacity, loss of natural motility after disassembly, and the need for modified host systems or auxiliary components for genome-editing applications (Zaidi and Mansoor, 2017).

### 2.5 Validation of transgene-free lines

How editing technologies are introduced, retained, and eliminated determines whether an edited plant is truly transgene-free, an important distinction for regulatory acceptance and commercial deployment (Bhattacharjee et al., 2023). The most widely used transformation technique, Agrobacterium-mediated transformation, requires T-DNA segregation, as demonstrated with examples discussed in the following sections. A fundamentally different route avoids foreign DNA altogether: using preassembled Cas9 or Cas12 complexes delivered directly into protoplasts, where they edit the genome and then get degraded, leaving no trace of foreign DNA (Woo et al., 2015). Other alternatives include transient Agrobacterium or non-integrating viral vectors, which allow *in planta* editing and tissue culture-free (Bhattacharjee et al., 2023). Finally, none of these approaches are self-verifying and still require explicit validation. The common practice for screening the absence of foreign DNA includes classic techniques such as PCR and Southern blotting. With the new sequencing technologies, it is recently common to start using whole-genome sequencing or deep sequencing targeting the on-target editing (Bhattacharjee et al., 2023).

## 3 Genetic engineering tools enabling synthetic biology

### 3.1 CRISPR-based systems

CRISPR (clustered regularly interspaced short palindromic repeats) loci and the protein components encoded by *Cas* (*CRISPR-associated*) genes are small RNA-based defense systems found in archaea and bacteria, which provide adaptive protection against foreign invading genetic elements, mainly phages and plasmids (Terns and Terns, 2011).

The CRISPR-Cas system comprises two major classes: class 1 and class 2 (Zhang D. et al., 2021). Class 1 systems contain 3 types: I, III, and IV, which include Cas3, Cas10, and Csf1 proteins (Makarova et al., 2015). Class 1 is a complex of Cas proteins that work together to bind and degrade DNA. On the other hand, class 2 has larger Cas protein that performs the same function as the multiple Cas proteins in class 1 (Zhang D. et al., 2021). Class 2 has 3 different types (II, V, VI) that contain (Cas9, Cas12a-e, and Cas14a-c) and (Cas13a-d) proteins, respectively (Makarova et al., 2015).

Class 2 CRISPR-Cas systems are widely used in genome editing due to their simplicity, requiring only two components: a single Cas protein and a single guide RNA (gRNA). The gRNA is formed by combining the spacer-containing CRISPR RNA (crRNA) and the transactivating crRNA (tracrRNA) into a single RNA molecule (Zhang D. et al., 2021). The mechanistic accuracy and programmability of CRISPR-Cas9 have enabled its development into versatile genome-editing tools, facilitating the improvement of traits such as increased yield and other desirable characteristics (Terns and Terns, 2011; Mishra et al., 2024). The major CRISPR-based technologies used in plant metabolic engineering, together with their representative delivery approaches and applications, are summarized in **Table 1**.

**Table 1:** Overview of CRISPR-based and transcriptome engineering technologies discussed in this review.

Technology	Mechanism	Delivery	Main application	Plant system
*CRISPR-Cas9 knock-out*	Cas9-induced double-strand break (DSB) repaired by error-prone NHEJ, producing indels	Agrobacterium, biolistic, protoplast delivery, viral vectors	Functional characterization/loss-of-function	Rice (Sedeek et al., 2023); Maize (Wu et al., 2023; Ma et al., 2024)
*Multiplex CRISPR-Cas9*	Simultaneous editing multiple genes/loci	Agrobacterium or biolistic; protoplasts	Rewiring metabolic pathways and modifying multiple pathway nodes	Tomato (Li et al., 2018, 2022); Wheat (Abdallah et al., 2025)
*Knock-in (HDR-mediated)*	Cas9-induced DSB repaired uding a donor template via HDR	Agrobacterium, protoplast; delivery plus donor DNA	Targeted gene or cassette insertion	Rice (Dong et al., 2020; Ali et al., 2026), *Nicotiana benthamiana* (Ali et al., 2026)
*CRISPRi*	dCas9 fused to repressor domain, targeted to a promoter	Agrobacterium, protoplasts	Transcriptional gene silencing without DNA cleavage	*Arabidopsis Thaliana* (Khan et al., 2025; no date); *Physcomitrium patens* (Khan et al., 2025); *Triticum aestivum* (Khan et al., 2025); *Brassica napus* (Khan et al., 2025); *Nicotiana benthamiana* (Karlson et al., 2022)
*CRISPRa*	dCas9 fused to transcriptional activation domain	Agrobacterium, protoplast	Transcriptional gene activation without DNA cleavage	*Arabidopsis Thaliana* (De Melo et al., 2020)
*Base editing*	Cas9 nicase or dCas9 fused to deaminase	Agrobacterium, biolistic, protoplast	Precise single-nucleotide conversion without DSBs or donor DNA	Maize (Kaul et al., 2024)
*Prime editing*	Cas9 nickase–RT, guided by pegRNA	Agrobacterium, protoplast	All 12 base to base conversion plus small insertion/deletion, without DSBs or donor DNA	Rice (Lou et al., 2025); tomato (Lou et al., 2025); *Salvia miltiorrhiza* (Yu et al., 2026)
*CRISPR-Cas13 (RNA editing or targeting)*	RNA-guided ribonuclease that binds and degrades complementary RNA	Viral vectors, Agrobacterium	Antiviral resistance/RNA-level gene knockdown	*Nicotiana benthamiana* (Aman et al., 2018); *Potato* (Zhan et al., 2023)
*R2 retrotransposon insertion*	RNA-template, target- primed reverse transcription at rDNA safe- harbor sites	Agrobacterium	DSB-free, kilobase cassette insertion	*Nicotiana benthamiana* (Ali et al., 2026), Rice (Ali et al., 2026)

The table summarizes the function, delivery methods, major metabolic engineering applications, and representative plant systems for commonly used CRISPR-based technologies.

### 3.2 Gene disruption (knock-out)

A common approach in genetic engineering is the disruption of gene function for functional studies or crop improvement. Prior to the development of advanced gene editing technologies, the most effective method for gene function studies involved introducing T-DNA into the genome via Agrobacterium, resulting in random insertion and disruption of one or more genes to generate mutant plants, which required subsequent screening for the gene of interest (Krysan et al., 1999; Alonso et al., 2003). This process is time-consuming and imprecise due to unpredictable insertion sites, which could affect multiple genes and leave residual foreign DNA in the genome. With new technologies, particularly CRISPR-Cas9, it is now possible to target genes with minimal off-target effects, making gene knockouts a routine and efficient method for gene characterization (Campenhout et al., 2019). CRISPR-Cas9 generates double-strand breaks (DSBs) that are repaired by Non-Homologous End Joining (NHEJ), an error-prone process (Li and Xia, 2020). That process frequently creates deletions or insertions (INDELS) that are used as an advantage in developing knock-out genes.

In maize, starch branching enzymes SBEI and SBEIIb are required for the production of the highly branched amylopectin (Ma et al., 2024). Ma et al., 2024 used CRISPR-Cas9 technology to target both genes and introduce a one-nucleotide insertion in *SBEI* and a four-nucleotide deletion in *SBEIIb*, causing a frameshift mutation (Ma et al., 2024). The mutant plants showed reduced starch-branching activity and increased amylopectin production, resulting in higher amylose accumulation. These changes in the metabolic pathway led to increased levels of resistant starch content (RSC) in the engineered maize lines (Ma et al., 2024).

Furthermore, Wu et al., 2023 produced independent *ZmSWEET1b* CRISPR knockouts. Lines exhibited decreased sucrose and fructose levels, leaf chlorosis, smaller ears and seeds, and increased wilting when exposed to salt stress, concurrent with downregulation of Na+-efflux genes *ZmSOS1* and *ZmH+-ATPASEs*, consequently establishing *ZmSWEET1b* as a positive regulator of salt-stress response and assimilate distribution (Wu et al., 2023).

In rice, Sedeek et al., 2023 used CRISPR-Cas9 to knock out three major suppressors of heading dates (*hd2*, *hd4*, and *hd5*) in the black Indonesian rice (Cempo Ireng). The T1 plants showed a significantly shorter life cycle and an earlier heading date, with flowering about 27–33 days earlier (Sedeek et al., 2023).

### 3.3 Multiplex CRISPR-Cas9 editing

Multiplex Cas9 editing, which enables the simultaneous targeting of multiple genes in a single transformation event, has become a standard approach for modulating metabolic flux. For example, a widely cited study on tomato used CRISPR-Cas9 multiplex editing of carotenoid-pathway genes, particularly stay-green 1 (*SGR1*), and found that lycopene levels in the most complete mutant line were approximately 5.1-fold higher than those in wild-type plants (**Figure 1**) (Li et al., 2018). Additionally, CRISPR-Cas9 knockout of the *Sl7-DR2* isoform of *7-dehydrocholesterol reductase* in tomato resulted in accumulation of the vitamin D3 precursor, 7-dehydrocholesterol, to up to 600 µg per gram of dry weight in leaves, with minimal impact on phytosterol or brassinosteroid biosynthesis (Li et al., 2022).

In Wheat, CRISPR-Cas9 knockout of conserved homologous sequences of *TaSal1* across multiple gene copies resulted in a significant improvement under polyethylene glycol (PEG)-induced stress. These knockout lines also maintained high germination rates, exhibited enhanced shoot and root development, and showed increased fresh and dry biomass (Abdallah et al., 2025).

### 3.4 Precise insertion (knock-in)

In plant synthetic engineering, inserting genes into the genome by methods other than standard *Agrobacterium tumefaciens*-mediated transformation system remains challenging. Alternative methods for inserting genetic material have been explored; however, in many plant species, the efficient and precise introduction of desired genes into specific genomic loci remains difficult. Targeted gene insertion using the CRISPR-Cas9 system is also limited in many plant species.

Dong et al., 2020 showed one of the best examples of Cas9-mediated gene knock-in, which enabled the insertion of a 5.2kb carotenoid cassette at a safe harbor in rice. Following the Golden Rice 2 (GR2) approach, the cassette includes two carotenoid biosynthetic genes: the phytoene desaturase (*CrtI*) gene from *Pantoea* (formerly *Erwinia uredovora*) fused to a chloroplast transit peptide from the pea RUBISCO small subunit (SSU) (*SSU-CrtI*), maize *phytoene synthase* (*ZmPSY)*, and a hygromycin resistance gene marker (Paine et al., 2005; Dong et al., 2020). Both structural genes are driven by the endosperm-specific glutelin promoter (Dong et al., 2020). The insertion was achieved via homology-directed repair (HDR) using the Cas9 machinery and a donor construct. Following segregation in the T1 generation, marker-free plants carrying the targeted insertion were obtained. This site-specific engineering of carotenoid genes with Cas9 enabled single-copy insertion and stable transformation, resulting in high β-carotene accumulation (~7.9 μg/g dry weight) in rice endosperm (Dong et al., 2020).

CRISPR-Cas9-mediated approaches remain constrained by factors such as the requirement for donor DNA templates, dependence on DNA repair pathways, and relatively low efficiency of targeted integration in many plant species (Mao et al., 2019). Recently, the *R2* retrotransposon, an RNA-mediated gene-integration tool, has evolved as a way to engineer a specific site within the genome (Luo et al., 2025). *R2* retrotransposons are naturally mobile genetic elements that can recognize specific target sites and precisely integrate new DNA copies through the target-primed reverse transcription (TPRT) mechanism into the 25S ribosomal DNA in plants (rDNA) (Luo et al., 2025; Muchenje et al., 2026). R2 proteins include reverse transcriptase (RT) domains similar to those in non-long terminal repeat (non-LTR) retroviruses, as well as endonuclease and DNA-binding domains within a single open reading frame (Luo et al., 2025; Muchenje et al., 2026).

Ali et al., 2026 were the first to utilize R2 retrotransposons to insert genes in specific sites in plants and created a method for the insertion of long sequences of DNA into a plant’s genome via RNA templates that is DSB-free. They were able to insert genes for GFP, recombinase landing pads, and resistance, as well as cassettes, into the rDNA of *Nicotiana benthamiana* and rice, with integration efficiencies of 75% for full-length GFP and 17% in rice callus (Ali et al., 2026). Presenting a promising insertion method of R2 retrotransposons for plant genome editing.

### 3.5 CRISPRi/CRISPRa

The inactive form of the CRISPR-Cas9 protein, termed deadCas9 (dCas9), retains its DNA-binding activity but lacks endonuclease activity and can be fused to transcriptional regulators to control gene expression without cleaving the DNA (Karlson et al., 2021). The engineered dCas9 can be linked to a sgRNA complementary to the gene promoter region and bound to a transcriptional effector that either activates a gene (CRISPR activator, CRISPRa) or represses a gene (CRISPR interference, CRISPRi) (Boettcher and McManus, 2015). CRISPRi commonly uses dCas9 fused to a repressor domain, such as Krüppel-associated box domain (KRAB), leading to efficient gene silencing when targeted to (0 to +500 nt) downstream of the transcription start site (TSS). In contrast, CRISPRa uses dCas9 fused to the commonly used VP64 activation domain, and the optimal activation site is (-400 to -50 nt) prior to the TSS (Boettcher and McManus, 2015).

A reversible CRISPRi platform was implemented to engineer repressible promoters with varying strengths, NOT (inverter; output is repressed in the presence of an input sgRNA-dCas9 complex and active in its absence) and NOR (logical negation of OR; output is expressed only when no sgRNA inputs are present) gates. This system was first demonstrated in *Arabidopsis thaliana* protoplasts and subsequently in stably transformed Arabidopsis plants (Khan et al., 2025). Layering NOR gates enabled the construction of OR, NIMPLY, and AND functions, and cross-species activity was confirmed in *Physcomitrium patens*, *Triticum aestivum*, and *Brassica napus* protoplasts, representing a compact, modular, and fully reversible plant gene circuit (Khan et al., 2025).

Similarly, CRISPRi was utilized in *Nicotiana tabacum* suspension cultures to silence the *cinnamate-4-hydroxylase* (*NtC4H*) gene, which encodes a key branching enzyme in the phenylpropanoid pathway, by targeting the promoter region with three sgRNAs (Karlson et al., 2022). Transcript abundance of *NtC4H* was reduced by ~0.44-fold; however, this led to higher accumulation of other flavonoid compounds due to competing genes in the phenylpropanoid pathway (Karlson et al., 2022).

A further example was shown by De Melo et al., 2020, who used CRISPRa-mediated transcriptional activation to activate drought-responsive transcription factors such as AREB1 and ABF2. Targeting the *AREB1* promoter with dCas9 fused to a histone acetyltransferase elevated *AREB1* expression and downstream activation of abscisic acid (ABA)-responsive gene networks, resulting in significantly enhanced plant tolerance to severe water deficit in Arabidopsis (De Melo et al., 2020).

### 3.6 Base editing

Base (BEs) and prime (PEs) editors facilitate precise nucleotide alterations without inducing DSBs, enabling more targeted modifications to enzyme performance, promoter function, and specific alleles while reducing off-target effects. However, editing effectiveness and delivery continue to vary across plant species and tissues, highlighting the need for further improvements in designing and expression strategies (Zhang et al., 2026).

Stable glyphosate tolerance in crop plants would greatly benefit crop production by providing an effective means of weed control. Kaul et al., 2024 reported stable glyphosate tolerance in *Zea mays*, achieved by introducing single amino acid substitutions into the 5-enolpyruvylshikimate-3-phosphate synthase (*EPSPS*) gene that plays a role in herbicide resistance. A CRISPR-Cas9 construct was co-delivered with an HDR donor template, resulting in targeted *EPSPS* editing in maize (Kaul et al., 2024). Edited T0 plants had single HDR-modified *EPSPS* alleles and exhibited robust glyphosate tolerance in greenhouse tests, with no negative effects on growth or agronomically important traits (**Figure 1**) (Kaul et al., 2024).

### 3.7 Prime editing

The prime editing (PE) technology consists of a fusion protein composed of a Cas9 H840A Nickase fused to a Moloney Murine Leukemia Virus (M-MLV) reverse transcriptase, along with the prime editing guide RNA (pegRNA) (Anzalone et al., 2019). PE is highly precise, allowing all possible 12 base-to-base conversions, targeted insertions, and targeted deletions without producing DSBs or requiring donor templates (Anzalone et al., 2019). In PE, Nickase Cas9 is fused to reverse transcriptase with a pegRNA modified to contain a primer binding site (PBS) and a designed reverse transcription template (RTT). Together, they cut a desired single strand (Anzalone et al., 2019). Following that, this specific strand will be incorporated into the DNA by the cell’s repair machinery (Anzalone et al., 2019). After its development, PE was successfully adapted for precise genome editing in crop species, including rice and wheat, demonstrating its applicability in plants (Lin et al., 2020).

Very recently, Lou et al., 2025 used prime editors to insert a 10-bp heat-shock element (HSE) into the promoters of cell-wall-invertase (CWIN) genes in elite rice and tomato varieties, a strategy named CROCS (climate-responsive optimization of carbon partitioning to sinks). Multilocation field trials showed tomato yield gains of 14-47% under normal conditions and rescued 56-100% of yield losses under heat stress; rice yields increased by 7-13% under normal conditions and by around 25% under heat stress, recovering up to 41% of heat-induced grain losses (**Figure 1**) (Lou et al., 2025).

For medicinal-plant metabolic engineering, Yu et al., 2026 developed MediPlant-NEPE, an expression-optimized prime editor for medicinal species. In *Salvia miltiorrhiza*, MediPlant-NEPE-mediated editing of *SmTCP15* produced biallelic mutants with deeper flower pigmentation and 1.54-fold increase in anthocyanidins (delphinidin and cyanidin), relative to wild-type (Yu et al., 2026).

### 3.8 RNAi-based gene silencing technology

RNA interference (RNAi) is a natural molecular process that triggers post-transcriptional gene silencing (PTGS) via double-stranded RNA (dsRNA), leading to the degradation of specific messenger RNAs and/or the suppression of gene expression (Younis et al., 2014). This pathway enables targeted knockdown of genes, making RNAi an effective tool for functional genomics and crop improvement by temporarily or permanently suppressing gene function without altering the underlying DNA sequence (Younis et al., 2014).

The CRISPR-Cas13 system for plant virus control was a significant breakthrough in targeting RNA. Aman et al., 2018 demonstrated that plant codon-optimized *Leptotrichia shahii* Cas13a (LshCas13a) produced in *Nicotiana benthamiana* with crRNAs targeting Turnip mosaic virus (TuMV) efficiently degraded viral RNA, lowering viral accumulation, GFP fluorescence, and disease symptoms. Additionally, they showed that Cas13a could convert precursor crRNAs into mature crRNAs, enabling multiplex RNA targeting from a single polycrRNA transcript (Aman et al., 2018).

Zhan et al., 2023 engineered Cas13d-mediated multiplex RNA targeting in *Solanum tuberosum* to confer broad resistance against multiple RNA viruses. CRISPR-Cas13 effector, belongs to class 2 type IV, is linked to a polycistronic tRNA-gRNA (PTG) expression system that enables simultaneous production of four virus-specific guide RNAs (Zhan et al., 2023). They successfully generated stable transgenic potato plants using the Cas13d-based PTG construct. The transgenic lines were verified to express Cas13d effector and produce the four virus-specific guide RNAs required for multiplex virus targeting. These PTG-expressing lines showed strong reductions in *Potato Virus Y*, *X*, S (PVY, PVX, PVS), and *Potato leaf roll virus* (PLRV) accumulation when each virus was individually inoculated, and they also maintained effective resistance against mixed infections involving two or three viruses (Zhan et al., 2023). The transgenic plants showed only slight disease symptoms and had lower viral copy numbers than wild-type and single-target lines (Zhan et al., 2023). These results, under synergistic mixed-virus infection conditions, reveal superior plant performance. Furthermore, this work establishes a compact, modular, and highly scalable technique to combat plant viruses by targeting their RNAs, and demonstrates a practical method for conferring broad-spectrum resistance to crops (Zhan et al., 2023).

### 3.9 Synthetic gene stacking and immune circuit engineering

Stem rust (SR), caused by the fungal pathogen *Puccinia graminis f.* sp. *Tritici*, is recognized as a serious threat to the major crop wheat, causing significant yield losses globally (Singh et al., 2015). Efforts have focused on identifying SR resistance genes to develop rust-resistant wheat varieties and on ensuring stable yields through approaches such as conventional breeding and genome editing, including gene stacking (Ellis et al., 2014). SR resistance genes encode members of an extremely polymorphic superfamily of intracellular nucleotide-binding leucine-rich repeat (NLR) receptors (Dangl et al., 2013). NLRs function within the cell and are essential for the defense and control of plant second-tier immune system (Dangl et al., 2013). Zhang J. et al. (2021) identified *SR26* and *SR61*, two *SR* genes, in tall wheatgrass (*Thinopyrum ponticum*), through Next-generation Sequencing (NGS), specifically mutational resistance gene enrichment sequencing (MutRenSeq) (Zhang J. et al., 2021). *SR26* and *SR61* genes encode a coiled-coil (CC) domain at the N-terminus, NB-ARC domain and LRR motifs at the C-terminus (CNL), which are immune receptors. Although *SR26* and *SR61* evolved from the same species, they share only 35% sequence identity (Zhang J. et al., 2021). However, through synthetic engineering, a recombinant chromosomal segment containing both *SR26* and *SR61* genes, which links the genes into a single introgression as a gene stack when transformed into wheat, showed greater resistance to SR (**Figure 1**) (Zhang J. et al., 2021). This result reveals that gene stacking is a viable strategy for enhancing pathogen resistance in crops.

### 3.10 Synthetic and edited promoters

Promoter activity is certainly one of the limiting factors in assembled multi-gene pathways. Recently, Amack, Ferreira and Antunes, 2023 systematically mutated the Cauliflower mosaic virus (*CaMV*) 35S TATA box and flanking sequences, generating a library of TATA-box variants that embrace a wide range of transcriptional expression. The library was delivered as Golden-Braid-compatible parts, providing a directly usable, reproducible set of tunable promoters for synthetic plant circuits (Amack et al., 2023).

New tools such as modular cloning frameworks (MoClo, GoldenBraid, MIDAS), based on Type IIS Golden Gate assembly, provide the physical substrate for stacking standardized parts. The original Engler-lab plant Golden-Gate toolbox and its derivatives underpin most multi-gene plant pathway projects today (Engler et al., 2014). CRISPRi-based logic gates introduce the regulatory layer necessary for truly programmable plant circuits (Khan et al., 2025).

In rice, Ludwig et al., 2024 employed CRISPR-Cas9 technology to excise a single ARR1AT *cis*-element at position -933 within the OsNAS2 promoter. The modification increased zinc concentration in T4-polished grain and enhanced yield-related parameters, therefore demonstrating a transgene-free possibility for zinc biofortification (Ludwig et al., 2024). A prior study by Johnson et al., 2011 showed that overexpression established an upper threshold: *OsNAS2* overexpression lines attained approximately four-fold enrichment in iron and two-fold enrichment in zinc in polished grain.

## 4 Integration with AI and machine learning

Beyond direct genome engineering, artificial intelligence is becoming increasingly prominent as a design tool. For example, Singh et al., 2025 describes a generalized platform for AI-powered autonomous enzyme engineering. The platform integrates machine-learning models and large language models with biofoundry automation, eliminating the necessity for human decision in iterative enzyme variant design-build-test cycles. Specifically, for plants, Xiang et al., 2026 developed an AI framework used to predict and design plant core promoters. The field is advancing toward closed-loop pathway optimization, wherein a model proposes designs and a biofoundry executes them (Singh et al., 2025). However, the use of AI in crop design remains limited due to restricted data and the inability of the models to generalize (Hao et al., 2025). The availability of large, high-quality omics datasets remains concentrated in a limited number of well-studied plant species, such as Arabidopsis and rice, which constrains the development and application of AI models across a broader range of crops (Yan and Wang, 2022). Deep learning models can function as “black boxes”, making it difficult to foresee how the model makes its predictions based on the data (Mostafa et al., 2023). Though AI has significant potential to accelerate crop engineering, its full potential can only be realized with large, standardized datasets combined with validated biological insights from experiments.

## 5 Future perspectives

Genetic engineering and synthetic biology strategies offer the potential to address global nutrient deficiencies, enhance crop stress tolerance, and facilitate sustainable production of high-value bioactive compounds (**Figure 1**). Using these novel systems, nutrient metabolism in crops can be reprogrammed while minimizing the risk of unintended secondary metabolic perturbations.

However, several difficulties remain. (i) Reproducibility across species is limited. Many components characterized in model species, such as Arabidopsis or *Nicotiana benthamiana*, perform unpredictably in crop species and thus require cross-species characterization. (ii) Compartmentalization and metabolon engineering-scaffold proteins, protein fusions, and organelle targeting can enhance yields but can introduce new problems. (iii) Off-target effects and pleiotropy are concerns when editing regulatory hubs. (iv) Regulatory systems for transgene-free edited plants are generally inconsistent. (v) The data layer: predictive artificial intelligence models require huge, standardized multi-omics datasets linked to controlled phenotypic readouts.

Pleiotropic and context-dependent effects typically arise because engineered genes or regulatory elements sit at metabolic branch points, sharing precursor pools or rate-limiting enzymes with other pathways. For example, engineering higher levels of anthocyanins in tomatoes demonstrates some of the trade-offs and pleiotropic effects of metabolic engineering (Cerqueira et al., 2023). Constitutive activation of ANT1 increases anthocyanin levels but reduces photosynthetic activity, slows vegetative growth, and produces fewer, smaller fruits, resulting in lower yield (Cerqueira et al., 2023). These results prove that improving a single trait is not enough; instead, an iterative design-test-learn cycle is needed, in which multi-omics and agronomic phenotyping tools identify problems and inform redesign.

Three trends appear to be the most significant moving forward: (a) the precise modification of *cis*-regulatory regions in crops through prime editing, exemplified (b) integrated plant and microbial production platforms, utilizing *N. benthamiana* and yeast; and (c) artificial intelligence-guided closed-loop design, build, and test processes across promoters, gRNAs, and enzymes.
